# Giant Bladder Stone: A Case Report and Literature Review

**DOI:** 10.31662/jmaj.2022-0061

**Published:** 2022-06-17

**Authors:** Tiopan Napitupulu, Martin Susanto, Grace Duma, Eka Yudha Rahman

**Affiliations:** 1 Department of Medicine, University of Sumatera Utara, Medan, Indonesia; 2 Department of Urology, University of Lambung Mangkurat, Banjarmasin, Indonesia

**Keywords:** giant, bladder, stone, case, report, literature, review

## Abstract

A urinary tract stone is a common urologic problem in Asia. In recent years, the incidence of urinary tract stones has increased but tends to be neglected by people with a lack of knowledge and low socioeconomic status. The occurrence of a bladder stone weighing more than 100 grams is unusual. We report the case of a 46-year-old Indonesian male patient who presented with complaints of lower abdominal pain, urinary pain, frequency, urgency, and hematuria. The patient also had severe anemia due to chronic hematuria. The patient then received open cystolithotomy because the ultrasonographic and X-ray findings of the kidney, ureter, and bladder revealed a giant bladder stone. This bladder stone measured 62 × 59 mm and weighed 301 grams. Gender, age, living in a tropical country, and lack of knowledge contributed to the patient’s neglect of symptoms.

## Introduction

A urinary tract stone is one of the most prevalent urologic diseases in Asia. The frequency, incidence, and composition of urinary tract stones vary around the world and have altered over the previous few decades, with the prevalence ranging from 7% to 13% in North America, 5% to 9% in Europe, and 1% to 5% in Asia. Urinary tract stones are prevalent in 5%-19.1% of the population in West Asia, Southeast Asia, South Asia, and some developed countries (South Korea and Japan), but just 1%-8% of the population in East Asia and North Asia ^(1), (2)^. Based on data from the Indonesian Ministry of Health, the incidence of urinary tract stones in the country in 2002 was 37,636 new cases, with 58,959 visits. The total number of patients treated was 19,018, with 378 deaths ^(3)^. Urinary tract stones can be found in many locations, such as the kidney, ureter, urethra, and bladder.

A bladder stone weighing over 100 grams is a rare occurrence ^(4)^. This case report presents the case of a giant bladder stone in a 46-year-old male patient, who complained of abdominal pain, urinary pain, frequency, urgency, and hematuria. The patient eventually received open cystolithotomy because of the massive size of the stone.

## Case Report

We report the case of a 46-year-old Indonesian male patient, who was admitted to the Pambalah Batung General Hospital, with the chief complaints of lower abdominal pain, urinary pain, frequency, urgency, and hematuria. The following symptoms were absent: fever, nausea, vomiting, nocturia, and urinary incontinence. From the history taking, it was known that the patient had passed a small stone when urinating about 5 years ago. The patient’s daily job was a farmer, and he had graduated junior high school.

On a physical examination, the patient’s vital signs were normal; abdominal palpation was within normal limits and the bladder was not palpable. An assessment of the patient’s radiological and blood laboratory examinations was also carried out.

**Figure 1.**
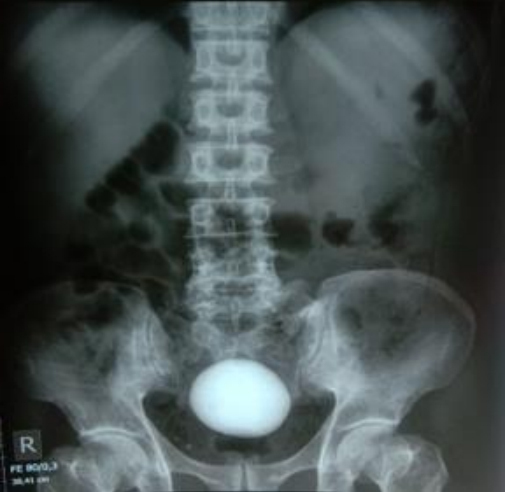
The patient’s bladder stone seen on an X-ray.

An X-ray of the kidney, ureter, and bladder (KUB) revealed the giant bladder stone shadow (Figure 1), and ultrasonography (USG) showed a bladder stone with chronic cystitis, bilateral hydroureters, and bilateral moderate hydronephroses.

Preoperative blood laboratory examinations showed severe anemia, with a hemoglobin value of 5 g/dL (Table 1). The patient received a transfusion of 6 packed red cell units before surgery, and after the transfusion, the hemoglobin value increased to 10 g/dL (Table 2). Although USG still showed signs of bladder inflammation, namely, thickening and irregularity of the bladder wall, the patient was not treated with antibiotics before surgery because the peripheral white blood cell counts were still normal. However, the urine culture examination was still being carried out, and the results were expected in 4-5 days. Renal function examination showed a mild increase in urea and blood urea nitrogen, but there was no indication of hemodialysis because of mildly impaired renal function (Table 1).

**Table 1.** Preoperative Laboratory Tests.

Hematology	Patient’s value	Reference value	Measurement unit
Hemoglobin	5.00	14.00-17.50	g/dL
-Hematocrit	14.2	40.00-50.00	%
-Mean corpuscular volume	73.8	80.00-96.00	fL
-Mean corpuscular hemoglobin	25.9	28.00-33.00	pg
Erythrocyte	1.93	4.50-5.90	×10^6^/μL
Leukocyte	8,400	4,400-11,300	/μL
Thrombocyte	639,000	142,000-424,000	/μL
**Renal function test**	**Patient’s value**	**Reference value**	**Measurement unit**
Urea	41	15-39	mg/dL
Blood urea nitrogen	19.10	7-18	mg/dL
Creatinine	1.0	<1.20	mg/dL

**Table 2.** Postoperative Laboratory Tests.

Hematology	Patient’s value	Reference value	Measurement unit
Hemoglobin	10	14.00-17.50	g/dL
-Hematocrit	29.2	40.00-50.00	%
-Mean corpuscular volume	79.2	80.00-96.00	fL
-Mean corpuscular hemoglobin	27.4	28.00-33.00	pg
Erythrocyte	4.2	4.50-5.90	×10^6^/μL
Leukocyte	9,200	4,400-11,300	/μL
Thrombocyte	356,000	142,000-424,000	/μL
**Renal function test**	**Patient’s value**	**Reference value**	**Measurement unit**
Urea	42	15-39	mg/dL
Blood urea nitrogen	20	7-18	mg/dL
Creatinine	1.3	<1.20	mg/dL

As the stone size was very large, open cystolithotomy was performed to remove the stone from the bladder. The operation was performed under general anesthesia and minimal blood loss was reported.

**Figure 2.**
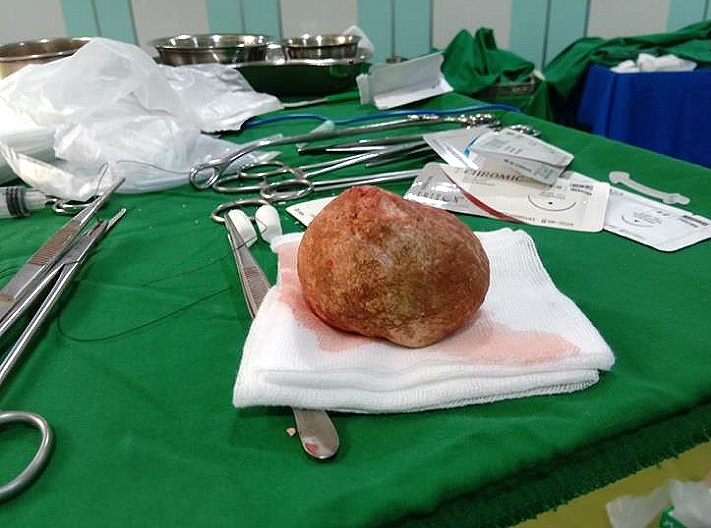
The patient’s giant bladder stone extracted by using open cystolithotomy.

As result, the giant bladder stone, measuring 62 × 59 mm and weighing 301 grams, was extracted intact from the bladder (Figure 2). Laboratory findings showed that the stone contained calcium oxalate. The later results of the patient’s urine culture showed the growth of *Proteus mirabilis* bacteria (Table 3), and the patient received an injection of Ceftriaxone 1000 mg every 12 hours. After surgery, the patient still complained of macrohematuria, which disappeared after 5 days. The patient was educated about the disease, the results of the operation, and the treatment given. He was discharged on the sixth postoperative day, and the indwelling catheter was then removed before discharge. The patient was asked to come for control to the surgical polyclinic. Subsequent assessments showed that the patient’s general condition was better and the patient acknowledged that all signs and symptoms had improved.

**Table 3.** Urine Culture Results.

Sample material	Urine	Description
Sample media	Sterile urine container	
Visual aspect		
Color	Yellow	
Microorganism count (CFU/mL)	<10^3^	>10^5^---------High UTI
		10^4^-10^5^-----Suspected UTI
		10^3^-10^4^----- Borderline; repeat the test
		<10^3^---------Contamination
Fresh specimen	Positive	
Gram-negative bacilli		
Colony from agar media	Positive	
Gram-negative bacilli		
Organism identification	*Proteus mirabilis*	

## Discussion

Epidemiological studies of urinary tract stones had shown a prevalence of between 1% and 19.1% in Asia; the highest incidence was found in the age group of 30-60 years ^(1)^. Bladder stones account for 5% of all occurrences of urinary tract stones and are caused by bladder blockage, neurogenic urinary dysfunction, urinary tract infections, or foreign substances ^(5), (6), (7)^. Adults with bladder stones are common in non-endemic areas. Men are more likely to be affected than women ^(5), (7), (8)^. Men are more likely to consume excessive alcohol and coffee and more meat than women. The testosterone hormone in men also increases the likelihood of stone formation, whereas estrogen in women seems to inhibit it ^(1)^. Otherwise, in endemic areas, children are also at high risk of developing bladder stones even without major anatomic abnormalities ^(7), (9)^. The main influencing factor is the climate (especially tropical or subtropical); lifestyle, lack of fluid intake, lack of education, and low socioeconomic factors also contribute to the formation of bladder stones ^(1), (9), (10)^. Bladder stones are frequent in Turkey, Iran, India, China, and Indonesia as a result of malnutrition in the early years of life; however, as social conditions improve, the incidence of bladder stones is decreasing ^(8)^. The main risk factors found in our patient was male gender, age 46 years, living in a tropical country, and lack of knowledge (he had graduated junior high school).

Bladder stones are characterized by recurrent urinary tract infections, hematuria, and urinary retention ^(5), (6)^. From history taking, we discovered the symptoms of lower urinary tract infection, lower abdominal pain, and gross hematuria in our patient. Bladder stones are mostly associated with kidney or ureteral stones and rarely occur without associated upper urinary tract stones, as was found in our patient ^(5)^. Primary bladder stones, which are made up of ammonium urate and calcium oxalate, are relatively frequent in Asia ^(8)^. After extraction, laboratory findings showed that our patient’s bladder stone did contain calcium oxalate.

The preferred method for diagnosis is cystoscopy, but an X-ray or ultrasound is sometimes sufficient ^(5), (6), (7)^. Endourologic equipment with a reliable energy supply has just been introduced. Endourologic therapy for bladder stones might be advised as a safe and effective noninvasive treatment option ^(11), (12)^. From January 2011 to April 2015, Deswanto et al. examined data from the medical records of 92 patients with bladder stones at Cipto Hospital Mangunkusumo. In these investigations, 49 patients with bladder stones measuring 2.5 ± 2.0 cm received extracorporeal shock wave lithotripsy (ESWL), with 46 patients (93.9%) remaining stone-free, and 33 patients with bladder stones measuring 4.2 ± 2.8 cm underwent intracorporeal lithotripsy, with a 97% stone-free rate. Sectio alta was only performed in 10 of 92 patients, with a 100% stone-free rate ^(11)^. According to the findings of Cicione et al., the stone-free percentage of ESWL in bladder stones ranged from 72% to 99%. In most cases, no anesthetic is required for the procedure. A urethral catheter is used to fill and empty the bladder, making stone localization and fragment clearance easier. However, around 17% of patients require cystoscopy to remove the pieces, and 10%-25% of patients require repeat therapy ^(12)^.

Cystolithotomy is an acceptable treatment for big bladder stones because of their size ^(5), (6), (7)^. USG and KUB X-ray assessments were performed on our patient and revealed a giant bladder stone; based on these considerations, cystolithotomy was finally performed. The size of the stone is the most significant point to consider when selecting a treatment method. Endourologic equipment with an effective energy source has recently been created, although it is difficult to take calculi larger than 2 cm. The patient’s general condition, signs, and symptoms improved after the surgery ^(11), (12)^.

## Conclusion

A giant bladder stone with minimal symptoms is a rare case and should be treated with cystolithotomy. A giant bladder stone, measuring 62 × 59 mm and weighing 301 grams, was extracted intact from the patient’s bladder. In this case, the patient developed severe anemia as a result of prolonged hematuria, which was neglected by the patient for about 5 years. Laboratory findings revealed that the stone was calcium oxalate, which is the most common compound found in giant bladder stones. Gender, age, living in a tropical country, and lack of knowledge contributed to the patient’s neglect of symptoms.

## Article Information

### Conflicts of Interest

None

### Acknowledgement

The authors thank PubMed, the DOAJ, and Cochrane for the accessibility and wide publication of the journals that were collected and analyzed in this study.

### Author Contributions

All authors contributed to the study conception and design, performed data collection, made substantial contributions to the analyses and interpretation of the data, and wrote this manuscript. All authors read and approved the final manuscript.

### Patient Consent

The patient had agreed and signed informed consent regarding publishing the case in an academic journal without exposing his identity.

### Approval by Institutional Review Board (IRB)

Not applicable
